# Optimizing Dental Resin-Based Composite Polymerization with Distance and Exposure Variables: Preliminary Study

**DOI:** 10.3390/ma19071390

**Published:** 2026-03-31

**Authors:** Anna Lehmann, Bolesław Barszcz, Kacper Nijakowski

**Affiliations:** 1 Department of Conservative Dentistry, Poznan University of Medical Sciences, 60-812 Poznan, Poland; annalehmann@ump.edu.pl; 2 Institute of Molecular Physics, Polish Academy of Sciences, 60-179 Poznan, Poland; boleslaw.barszcz@ifmpan.poznan.pl

**Keywords:** resin-based composites, polymerization, degree of conversion, light-emitting diode, exposure mode, composite restoration

## Abstract

Resin-based composites (RBCs) are widely used in restorative dentistry, and their clinical performance depends on the degree of conversion (DC). Light-curing units are used to initiate polymerization, but factors such as the distance between the light source and the composite surface as well as different exposure modes can affect DC. This study aimed to evaluate the effect of curing distance on the DC of resin-based composites under different polymerization modes. Specimens of a standardized resin-based composite were prepared and cured using a light-emitting diode (LED) curing unit at varying distances (0 mm, 2 mm and 4 mm). Three exposure modes were applied: standard, soft-start, and pulse. The DC of the cured composites was determined using Raman scattering spectra measurements. The DC differed significantly depending on the evaluated factors and the measurement location within the samples (top vs. bottom). For measurements taken at the top of the samples, a highly significant effect of material type on the degree of conversion was observed (*p*-value < 0.001). Distance also showed a statistically significant influence (*p*-value = 0.049), whereas exposure mode did not significantly affect DC at the top surface (*p*-value = 0.391). Both curing distance and exposure mode significantly influence the polymerization efficiency of resin-based composites. Minimizing the distance between the light source and composite surface improves the DC, and selecting an appropriate exposure mode can partially compensate for increased distance. Clinicians should consider these factors to optimize the mechanical properties and longevity of composite restorations.

## 1. Introduction

Resin-based composites (RBCs) are among the most commonly used materials for direct tooth restorations [1]. Their well-known advantages, such as ease of handling, strong adhesion, and excellent aesthetic qualities, are counterbalanced by certain limitations, including polymerization shrinkage and the potential release of toxic or allergenic monomers [2,3]. For many years, extensive research has focused on evaluating the long-term stability of the polymer network under the challenging conditions of the oral environment [4,5]. Increasing attention has been directed toward the fact that the physicochemical properties of composite materials depend not only on their chemical composition but also on the techniques employed during placement and polymerization [6]. These materials are highly technique-sensitive, and even minor deviations during the restorative procedure may lead to a compromised filling, reduced mechanical performance, or an increased risk of pulpal irritation and postoperative complications [7]. An undisputed key indicator of the quality and performance of RBCs is the degree of conversion (DC)—that is, the percentage of carbon–carbon double bonds in the monomer that are converted into single bonds during polymerization. The DC directly influences the physical and biological properties of the final restoration, including hardness, strength, polymerization shrinkage, color stability, and biocompatibility [8,9].

The process of RBC polymerization is influenced by numerous factors, including the composition and optical properties of the resin (type of monomers, fillers, and pigments), the method of application, and the parameters of the curing light, such as light intensity (irradiance), exposure duration, polymerization mode, and, critically, the distance between the light-curing tip and the composite surface [10].

Numerous studies have shown that increasing the distance between the light source and the composite surface may adversely affect the quality of polymerization, particularly in deeper or less accessible areas of the restoration [11]. Furthermore, the quality of polymerization also depends on the curing mode, including the type of light source (LED or halogen), irradiance, exposure time, and polymerization protocol (e.g., incremental vs. bulk-fill techniques). In clinical practice, the curing light tip is often positioned at a certain distance from the composite surface rather than in direct contact. This increased separation may result in incomplete polymerization, which can compromise the longevity of restorations and lead to defects such as porosity, reduced hardness, greater polymerization shrinkage, monomer release, and even microleakage [12]. Moreover, increased distance from the curing light tip correlates with altered post-gel volumetric shrinkage and non-uniform shrinkage strain distribution within the RBC [13].

Despite the clear recommendations to polymerize the material from the shortest possible distance, there are clinical situations in which this is not feasible. This primarily applies to Class II cavities located on the proximal surfaces of posterior teeth. The depth of these cavities often ranges from 3 to 6 millimeters, with the additional height of the surrounding cusps (1–2 mm) further increasing the distance. As a result, the resin-based composite can be polymerized from a distance greater than 4–6 mm [14]. Until recently, it was believed that the main mechanism predisposing Class II RBC restorations to secondary caries was microleakage at the margins—particularly at the gingival margin, which often lacks sufficient enamel to ensure optimal adhesion due to tooth anatomy [15]. Technique-related factors were also considered important, including the quality of cavity preparation, the effectiveness of isolation, and the precision of matrix and wedge placement. Patient-related factors, such as the consumption of simple sugars and oral hygiene habits—especially interdental hygiene—should also be taken into account. Scientific studies have clearly demonstrated that composite restorations on interproximal surfaces are significantly more susceptible to secondary caries than those on other surfaces [16,17,18]. Unfortunately, there is still no clear evidence in the literature that the high incidence of secondary caries in Class II restorations may be related to the low conversion rate of the composite material used. Naturally, under clinical conditions, conducting a study that could demonstrate a direct relationship between secondary caries and this single factor is practically impossible.

In response to these clinical challenges, curing light manufacturers have introduced various exposure modes. Research has indicated the advantages of the soft-start mode over other curing protocols. According to the literature, the soft-start mode reduces polymerization shrinkage stresses compared with the standard mode, thereby limiting the formation of marginal gaps and improving the seal and mechanical integrity of the restoration [19,20]. However, our research has shown that soft-start mode was the least resistant to increasing distance [21]. The type of composite also influences the polymerization behavior. There is still no clear consensus in the scientific literature regarding the optimal polymerization protocol for flowable or bulk-fill resin-based composites [22,23]. In this study the authors analyzed three completely different materials, differing in the type of resin and filler as well as clinical indications, in order to assess the trend in DC and potential correlation with the irradiation mode.

Despite extensive research on resin-based composite polymerization, most previous studies have evaluated either curing distance or exposure mode independently, often focusing on a single composite type. Limited evidence is available regarding the combined influence of curing distance, exposure mode, and material composition on the degree of conversion at different depths within the restoration. Moreover, although inadequate polymerization has been suggested as a potential contributing factor to clinical failure in deep posterior restorations, no consensus exists regarding the optimal curing strategy under realistic clinical conditions where direct contact between the light-curing unit and the composite surface is not always achievable. Addressing this gap may provide clinically relevant guidance for optimizing polymerization protocols in challenging restorative scenarios.

Therefore, this study aimed to evaluate the effect of the distance between the curing light source and the composite surface on the degree of conversion of various RBCs under different polymerization exposure modes. We hypothesize that factors such as distance, type of exposure, and the type of composite material influence the degree of conversion depending on the surface of the sample. This investigation seeks to identify optimal clinical parameters for light curing that ensure reliable and durable composite restorations while maintaining optimal polymerization efficiency under realistic clinical conditions.

## 2. Materials and Methods

### 2.1. Tested Materials and Light-Curing Protocols

In this in vitro study, three dental restorative materials were used. Detailed characteristics of the materials are presented in Table 1.

Three types of light-curing protocols were applied. A total of 27 experimental groups were created, consisting of three types of RBCs, each cured using three different exposure modes and at three distances (Table 2).

### 2.2. Specimen Preparation

All specimen preparation was performed by a single operator to minimize variability. A total of 81 composite specimens (4 mm in diameter and 2 mm in height) were prepared, with three samples per experimental group. All materials, kept at room temperature, were inserted into the mold and intentionally overfilled. The material was covered with Mylar tape and pressed down with a metal spatula to even out the surface and remove excess material. Additionally, the Mylar tape prevented the formation of an oxygen-inhibition layer. First, samples of the three materials were prepared and exposed at a distance of 0 mm. Subsequently, additional samples were prepared and exposed at a distance of 2 mm. Finally, samples were prepared and exposed at a distance of 4 mm. Each sample was replicated three times. Each specimen was light-cured for 20 s using one of the three curing models, according to the manufacturer’s instructions (Woodpecker LED.H, Guilin Woodpecker Medical Instrument Co., Guilin, China). Three light-curing models were used in the study:

A. Standard mode, LED works in full power, 1000 mW/cm^2^.

B. Soft start mode, LED turns from weak to stronger and reaches the highest power in 5 s.

C. Pulse mode, LED pulsating between 850—1000 mW/cm^2^ in 2 s intervals.

The light-curing procedure was repeated while gradually increasing the distance between the light-curing unit (LCU) and the composite surface. The LCU was fixed in a holder, and the distance to the mold was adjusted using spacer rings to achieve 0, 2 and 4 mm separations. The lamps were fully charged and placed on the charger to maintain the maximum charge. Light irradiance measurements were made using a photometer Bluephase Meter II (Ivoclar Vivadent, Glattpark, Switzerland), with measurement range from 300 to 4000 mW/cm^2^.

After polymerization, the lower surface of each specimen was marked, and all samples were stored in light-proof containers filled with distilled and deionized water at 37 ± 1 °C for 24 h to complete the post-polymerization process.

### 2.3. Degree of Conversion Analysis

The convenient way for the determination of conversion degree (DC) in methacrylate materials is to use the relative intensities of bands at 1639 cm^−1^ and 1609 cm^−1^ observed in Raman spectra [24]. Those bands are related to aliphatic and aromatic CC stretching vibrations—the 1639 cm^−1^ band is due to -CH=CH_2_ stretching vibrations, which changes drastically in intensity with the polymerization of sample, while the 1609 cm^−1^ band is treated as an internal standard because its intensity is conserved during the process.

The Raman spectra of the samples were measured in a backscattering geometry using a Labram HR 800 spectrometer (Horiba Jobin Yvon, Villeneuve d’Ascq, France) with a He-Ne laser (λ = 632.8 nm) as an excitation source and a liquid-nitrogen cooled CCD detector. For calibration the Si line at 520.7 cm^−1^ was used. The laser beam was focused on the top and bottom of the sample using a ×50 objective. To prevent local overheating and thermally induced changes the power of the laser beam at the sample was reduced to about 0.9 mW. The single-spectrum acquisition time was 20 s and the spectrum of every sample (from top and bottom surface) was determined after averaging 40 accumulations. The spectra were recorded in the 1550–1800 cm^−1^ spectral region at room temperature (22 °C) and the spectral resolution was less than 2 cm^−1^.

The obtained Raman spectra were fitted and deconvoluted using Fityk 1.3.1 software [25]. First the simple 2-point background was subtracted from the spectra in the regions 1575–1660 cm^−1^ (for bulk-fill samples) and 1590–1655 cm^−1^ (for the rest). Next, the spectra were fitted using the Lorentzian peak shape function, assuming 4 (for bulk-fill samples) or 2 peaks in the region. All the functions were fitted to the experimental data using the Levenberg–Marquardt algorithm as implemented in Fityk 1.3.1 software. For calculation of conversion degree the integral intensity (area) ratio was used according to the equation

DC (%) = (1 − I_s_/I_p_) × 100,

where

I_s_ = I_1639_/I_1609_

I_1639_, I_1609_—integral intensities (area) of the bands observed at 1639 and 1609 cm^−1^ in the spectra of the sample.

I_p_ = I_p1639_/I_p1609_

I_p1639_, I_p1609_—integral intensities (area) of the bands observed at 1639 and 1609 cm^−1^ in the spectra of the pure (uncured) reference appropriate for specific samples.

### 2.4. Statistical Analysis

The determined degrees of conversion were analyzed using a three-way analysis of variance (ANOVA) in the model with the interaction. Two independent ANOVA models, separately for top and bottom surfaces of samples, were applied, including the following factors: material type (3 levels), exposure mode (3 levels), and distance from the curing tip (3 levels). All subgroups met the assumptions in accordance with statistical requirements. For the post hoc analyses the Tukey test was used. The significance level was defined as α = 0.05. The statistical analysis was performed using Statistica 13.3 (Statsoft, Cracow, Poland).

## 3. Results

**Figure 1:**
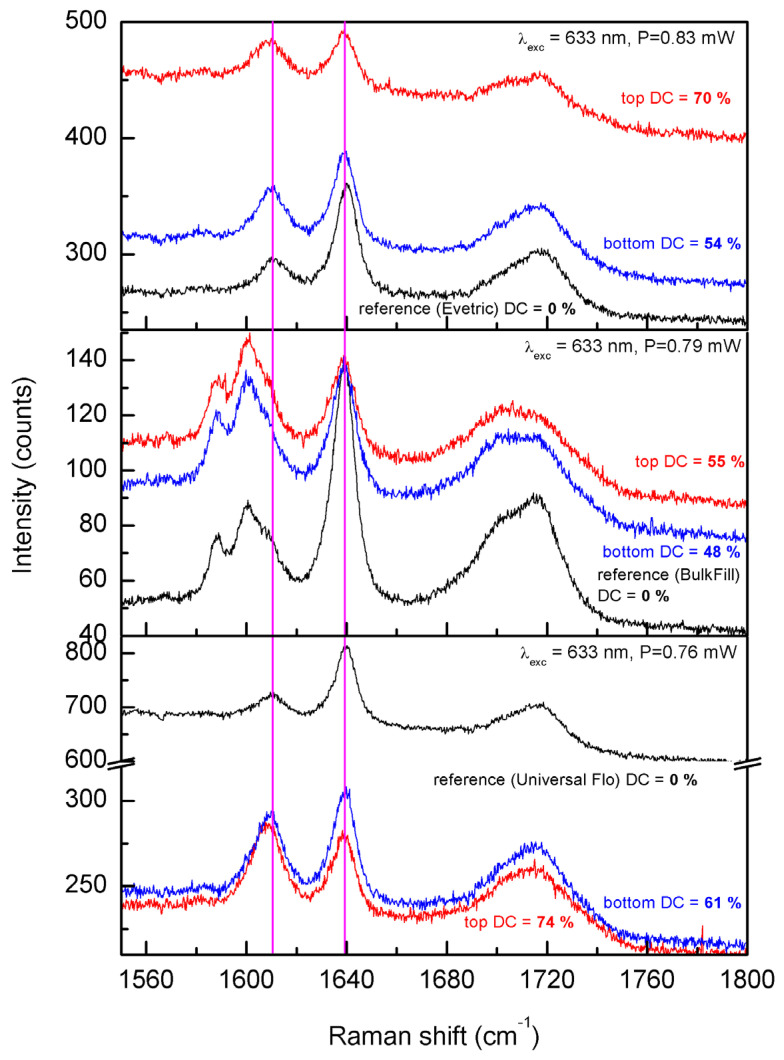
The representative Raman scattering spectra of the cured (red and blue) and uncured (black) samples prepared with Evetric (**top panel**), BulkFill (**middle panel**), and Universal Flo (**bottom panel**). The vertical magenta lines indicate the positions of the bands used for calculations of the conversion degree—1639 cm^−1^ (aliphatic CC stretching) and 1609 cm^−1^ (aromatic CC stretching). Note that for the Bulk Fill sample the 1609 cm^−1^ band is a component of the larger spectral feature. The deconvolution of the spectra from the middle panel are presented in Figure 2.

**Figure 2:**
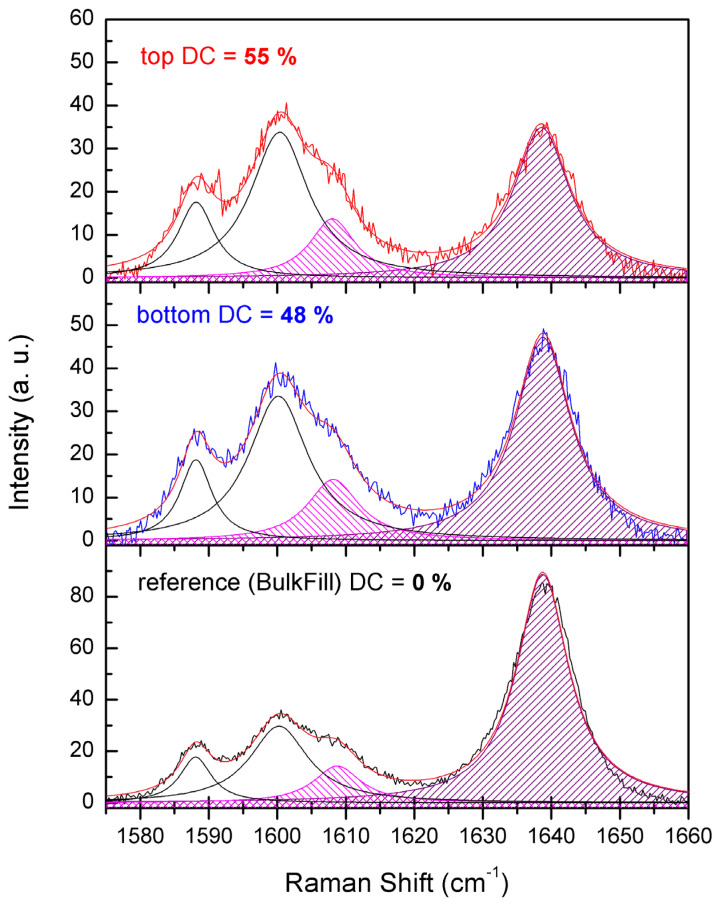
The deconvolution of Raman scattering spectra from the middle panel of Figure 1. The spectra were deconvoluted using 4 Lorentzian peaks (see details in text and in Table S2 in Supplementary Materials). The filled peaks were used for calculations of the conversion degree.

The representative spectra of the cured and uncured samples of all three composites are presented in Figure 1 and the exemplary deconvoluted Raman spectra are presented in Figure 2.

**Figure 3:**
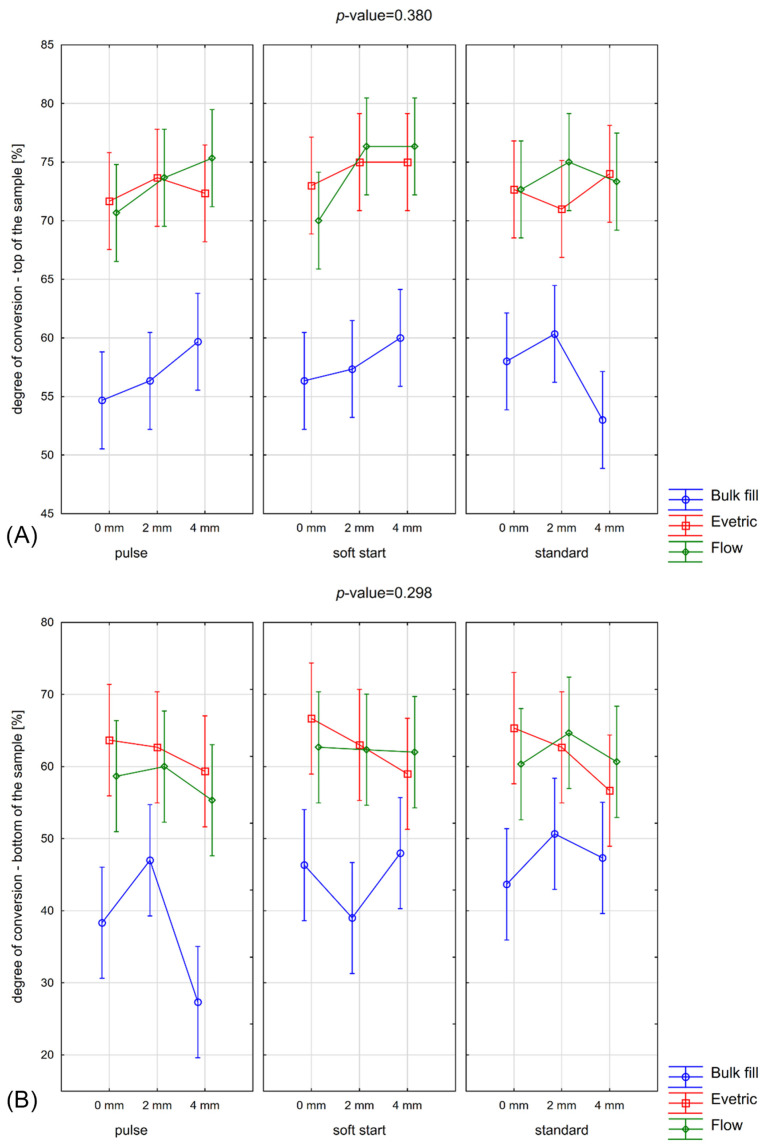
Comparison of the degree of conversion at the top (**A**) and bottom (**B**) of the samples depending on the interaction between type of material (Bulk fill, Evetric, Flow), exposure mode (pulse, soft start, standard), and distance (0 mm, 2 mm, 4 mm). Vertical bars indicate 95% confidence intervals.

The DC differed significantly depending on the evaluated factors and the measurement location within the samples (top vs. bottom). Detailed comparisons are presented in Figure 3 and summarized statistically in Table 3 and Table S1. For measurements taken at the top of the samples, a highly significant effect of material type on the degree of conversion was observed (*p*-value < 0.001). Distance also showed a statistically significant influence (*p*-value = 0.049), whereas exposure mode did not significantly affect DC at the top surface (*p*-value = 0.391). None of the two-way or three-way interactions reached statistical significance for top measurements.

At the bottom of the samples, material type again had a significant effect on DC (*p*-value < 0.001). In contrast to the top surface, exposure mode significantly influenced DC at the bottom (*p*-value = 0.032), while distance did not reach statistical significance (*p*-value = 0.068). Similarly, no significant interactions between material, exposure mode, and distance were detected for bottom measurements.

Across all experimental conditions, the degree of conversion was generally higher at the top of the samples compared with the bottom. This trend was consistent regardless of material type, exposure mode, or irradiation distance. The absence of significant interaction effects indicates that the influence of each factor on DC was independent and did not differ substantially between the top and bottom surfaces (Figure 3).

Tukey’s post hoc test revealed numerous statistically significant pairwise differences, primarily between different material types, confirming the dominant role of material composition in determining DC values. Differences related to distance and exposure mode were less pronounced and were mainly observed in top-surface measurements. Comparisons within the same material across varying exposure modes and distances frequently did not reach statistical significance, particularly at the bottom surface (Table S1).

## 4. Discussion

In a previous study, the authors focused on the analysis of the amount of light reaching the exposed surface from the lamp, considering various exposure modes and variable distance [21]. The study showed that increasing the distance most strongly affected the soft mode, causing a significant decrease in light irradiance measured by the photometer. We can assume that with an output energy above 1000 mW/cm^2^, in the standard mode, approximately 996 mW/cm^2^ reaches the sample at a distance of 2 mm, and after increasing the distance to 4 mm this value becomes 906 mW/cm^2^. For the soft start and pulse modes, the data were: 985 and 884 mW/cm^2^, and 993 and 893 mW/cm^2^, respectively.

However, that study analyzed only the amount of light energy reaching the radiometer surface. In dental practice, the depth of cavities varies, ranging from fractions of a millimeter to several millimeters [26]. The thickness of a composite layer is typically 1–2 mm or more (layers of 4 mm for bulk fill), and clinicians almost never position the curing light directly at a 0 mm distance due to the tooth anatomy [27]. Taking these variables into account, we designed a new study aimed at providing a deeper insight into the sample and creating a three-dimensional map of RBC polymerization. We employed Raman spectroscopy, as it is a well-established method for determining the degree of conversion. This technique offers high precision and versatility, and it is particularly valuable due to its ease of use and capability for in situ and depth-profiling measurements [28].

The discussion refers to the results analyzing the degree of conversion obtained not only by Raman spectroscopy but also by Fourier transform infrared spectroscopy (FTIR). Both methods are based on monitoring the same functional group by observing the reduction of C=C double bonds during polymerization. They offer complementary advantages and limitations [29,30].

The number of samples used for Raman analyses varies among studies and typically ranges from 50 to 150, with an n value of 3 to 5 per group [31,32,33,34]. It is generally accepted that 3–5 samples are sufficient to estimate measurement variability. Many studies to date have examined exposure modes primarily in terms of LED power, comparing low-power and high-power settings [35,36]. Polymerization performed with an advanced LED curing unit over a shorter exposure time (e.g., 3 s) has been shown to produce variable polymerization efficiencies across different types of composite resins. To achieve more homogeneous polymerization, extending the exposure duration to 10 s or longer to 20–40 s is recommended [37,38]. Therefore, in our study, we employed three exposure modes commonly used in commercial LED light-curing units (LCUs). The power and exposure time values adopted in this study (1000 mW/cm^2^ for 20 s) align with current clinical recommendations [39,40].

Numerous studies have shown that increasing the distance between the curing light and the composite surface beyond 4 mm results in insufficient polymerization. Therefore, we focused on distances of 0, 2, and 4 mm, which realistically reflect clinical conditions [11,36,40].

In this study, three commonly used composite materials with clearly different compositions and clinical indications were evaluated: an aesthetic nanohybrid composite typically used for anterior restorations (Evetric), a bulk-fill composite designed for posterior teeth (Filtek One Bulk Fill), and a multipurpose flowable composite (G-aenial Universal Flo). The flowable resin-based composite achieved the highest degree of conversion at the top of the specimen, reaching 74%. At the bottom of the sample, the DC exceeded 60%, which represents a very favorable outcome and is consistent with findings reported by other authors [41,42]. In contrast, the bulk-fill composite exhibited the lowest conversion among all tested materials. Although the top of the specimen achieved a DC above 55%, which is generally considered sufficient, the DC at the bottom did not exceed 45%. Numerous studies on various composite resins suggest that the optimal monomer conversion typically ranges between 52% and 75% [40,43,44]. However, the literature currently lacks a clearly defined, clinically acceptable minimum threshold for conversion [23]. Nevertheless, it has been suggested that conversion values below 55% may be insufficient for occlusal surfaces subjected to high masticatory loads [22]. As noted by Jakupovic, achieving a high DC in bulk-fill composites is particularly critical due to their increased layer thickness, which may reach up to 4 mm; therefore, special attention should be paid to ensuring adequate polymerization at greater curing distances [45]. Our findings confirm Jakupovic’s observations, as Filtek One Bulk Fill showed the greatest sensitivity to increasing distance from the LED light-curing unit. At the bottom of the specimen, the DC did not reach the suggested minimum value of 55%. It has been demonstrated that increasing irradiation time may enhance the polymerization process by prolonging the propagation phase of free radicals, which can contribute to a higher DC in light-cured resin composites [46,47]. Moreover, the study by Gömleksiz demonstrated that double exposure of a composite, even from an increased distance, can result in a higher DC than a single 20 s exposure applied close to the surface [40]. This finding represents a valuable and practical recommendation for clinical application.

For several years, researchers have been analyzing the differences in polymerization reactions under the influence of various exposure modes. The original standard mode is characterized by rapid free radical generation, resulting in faster polymer cross-linking, but it also results in internal stress. The soft-start mode exhibits a slower rate of polymerization initiation. This results in a longer pre-gel period, allowing for stress relaxation. However, the final DC depends on the subsequent exposure phase. It seems reasonable to first expose the sample using the standard mode and then supplement it with the soft-start mode. The pulse mode exhibits similar effects to the soft-start mode, also providing time for stress relaxation thanks to interruptions in energy emission [22,48,49]. It should be noted that a higher filler particle content limits the mobility of the monomers and can hinder full cross-linking [50]. Composites containing finer particles may have a smaller interparticle spacing, which promotes better light penetration and, therefore, a potentially higher conversion rate. At the same time, irregular or mixed shapes can affect light scattering and the final DC [51]. Our research confirms the results obtained by previous researchers. The Flow material tested, containing the least filler, had the highest DC, while Bulk Fill had the lowest DC. Evetric, containing mixed particles, yielded intermediate results, which may be related to the aforementioned increased light scattering.

The soft-start mode proved to be the most effective for polymerizing the composite at the top surface of the specimen and was only slightly inferior to the standard mode at the bottom surface. The DC values were appropriate and reached 69% at the top and 56% at the bottom in the soft-start mode, compared with 67% and 57%, respectively, in the standard mode. These findings confirm our previous observations regarding the influence of exposure modes at increasing curing distances [21]. We demonstrated that increasing the distance between the curing light and the composite surface by 2–4 mm resulted in a sufficient DC at the top of the specimen, reaching nearly 70% at 2 mm and approximately 60% at 4 mm. These results are consistent with those reported by other researchers [52]. The lower conversion observed at a 0 mm exposure distance may be related to a mismatch between the diameter of the composite specimen and that of the LED light tip. The pulse mode, even at increased distances from the irradiated surface, provided acceptable light intensity sufficient for effective polymerization at the top of the specimen. However, DC values obtained with this mode were the lowest among all tested exposure protocols. Consequently, this mode may be considered for use or recommended with caution in deep cavities with vital pulp, as it may reduce the risk of pulp overheating [53,54].

Among the tested materials, the flowable RBC exhibited the highest DC values. G-aenial Universal Flo contains TEGDMA and a low-viscosity dimethacrylate monomer (BisMPEPP), which belongs to the BisEMA-type monomer family. Previous studies have shown that the incorporation of lower-viscosity monomers into resin composites may enhance resin matrix mobility and facilitate free radical diffusion during polymerization, which can promote a higher degree of conversion [39]. Similarly, Yu et al. reported that resin systems characterized by lower initial viscosity and greater molecular mobility may achieve higher conversion levels due to improved radical propagation and reduced light scattering within the material [52]. However, since the exact monomer ratios in the investigated material are not disclosed by the manufacturer, the present results should not be interpreted as evidence of a direct effect of a specific monomer. Instead, the higher DC observed for this material may be associated with the overall characteristics of its monomer system and viscosity, which can influence polymerization efficiency.

Evetric also achieved relatively high DC values, although lower than those observed for G-aenial Universal Flo. This difference may be attributed to the higher molecular weight of the monomers used in Evetric. As reported by Gömleksiz, monomers with higher molecular weight exhibit reduced mobility, which may limit the final DC of the composite [40]. The addition of UDMA further benefits this material, providing greater flexibility compared to Bis-GMA systems. This improves fracture resistance and allows the composite to better absorb masticatory forces without brittle fracture [55]. Considering the wide variability in composite composition and behavior, it appears necessary to develop material-specific polymerization protocols tailored to the individual properties of each resin-based composite [35].

### Limitations and Future Directions

The present study has several limitations that should be acknowledged. As an in vitro investigation, it cannot fully replicate the complexity of the oral environment. Clinical factors such as the operator’s technique, patient-related variables, the presence of saliva, fluctuations in pH, and intraoral temperature variations may all influence polymerization outcomes and were not reproduced in this experimental design. In addition, only curing distances of 2 mm and 4 mm were evaluated; however, in clinical practice, the depth of posterior restorations may exceed 4 mm, which should be considered when interpreting the results.

The sample size was intentionally small, consistent with a preliminary study design, and no a priori power analysis was performed, limiting the statistical power and generalizability of the findings. Due to the pilot nature of the study and the small sample size (*n* = 3 per group), formal normality and variance tests have very limited statistical power and may not provide reliable conclusions. For this reason, the analysis should be interpreted as exploratory, aiming to identify potential trends in the influence of the investigated factors rather than to provide definitive statistical inference. Radiant exposure was not directly quantified at each curing distance and mode, and only a single increment thickness was assessed. Also, a study limitation may be the fact that without using a rigid surface during curing, the flexible strip may allow oxygen diffusion, creating a partially inhibited zone at regions with poor contact, affecting the top surface conversion. Furthermore, the physicochemical and mechanical properties of the materials were not evaluated, and the degree of conversion was not correlated with outcomes such as microhardness or flexural strength. Potential bias related to Raman spectral fitting parameters cannot be excluded, and no thermal measurements or aging procedures were conducted to assess long-term material stability.

Future studies should therefore include larger sample sizes with appropriate power calculations, evaluate thicker (e.g., 4 mm) bulk-fill increments, directly measure delivered radiant exposure under all curing conditions, correlate degree of conversion with mechanical and thermal properties, and incorporate aging protocols to better reflect long-term clinical performance.

## 5. Conclusions

Within the limitations of this study, the findings demonstrate that:

▪ Material composition is the primary determinant of degree of conversion.
▪ Curing distance significantly affects polymerization efficiency, particularly at the top surface.
▪ Exposure mode influences polymerization behavior at the bottom surface.
▪ Flowable composites achieved the highest degree of conversion across conditions, whereas bulk-fill material showed greater sensitivity to increased curing distance.

These findings provide experimental evidence supporting the selection of material-specific and distance-adjusted curing protocols in clinical practice.

### Clinical Significance

This study confirms that the polymerization efficiency of resin-based composites depends on material composition, curing protocol, and the distance from the LCU. The use of a flowable liner in deep cavities, combined with a standard curing mode, appears to provide the highest degree of conversion. Due to the relatively low degree of conversion observed, clinicians should exercise caution when using bulk-fill materials in thick increments. Future studies should investigate the impact of insufficient polymerization on the mechanical and biological properties of these materials, and clinical trials are warranted to confirm these in vitro findings under real-world conditions.

## Figures and Tables

**Table 1:** Detailed characteristics of the dental restorative materials used in the study.

Material	Material Group	Manufacturer	Composition	Lot Number
Filtek One Bulk Fill Restorative, Shade A2	Nano-composite	3M/ESPE, Seedfeld, Germany	Organic matrix: AUDMA, UDMA, 1,12-dodecane-DMA 20 nm silica; Filler fraction (wt%/vol%) 76.5/58.4, Fillers: 4–11 nm zirconia, ytterbium trifluoride filler consisting of agglomerate 100 nm particles	11670118
Evetric, Shade A2	Nano-hybrid composite	Ivoclar Vivadent, Schaan, Liechtenstein	Organic matrix: Bis-GMA, Bis-EMA, UDMA; Filler fraction (wt%/vol%) 80–81/55–57, Fillers: barium glass, ytterbium trifluoride, mixed oxide, copolymers (size 40–3000 nm)	Z0939B
G-aenial Universal Flo, Shade A2	Flowable composite	GC Europe, Leuven, Belgium	Organic matrix: Urethanedimethacrylate, Bis-MEPP, TEGDMA; Filler fraction (wt%/vol%) 69/50, Fillers: silicon dioxide, strontium glass, pigment (size 16–200 nm)	2501081

**Table 2:** Curing protocols used in the experimental subgroups.

Group	Resin-Based Composite	Curing Protocol	Distance	Sample Size *n*
1	Bulk fill	standard	0 mm	3
2	Bulk fill	standard	2 mm	3
3	Bulk fill	standard	4 mm	3
4	Bulk fill	soft start	0 mm	3
5	Bulk fill	soft start	2 mm	3
6	Bulk fill	soft start	4 mm	3
7	Bulk fill	pulse	0 mm	3
8	Bulk fill	pulse	2 mm	3
9	Bulk fill	pulse	4 mm	3
10	Flow	standard	0 mm	3
11	Flow	standard	2 mm	3
12	Flow	standard	4 mm	3
13	Flow	soft start	0 mm	3
14	Flow	soft start	2 mm	3
15	Flow	soft start	4 mm	3
16	Flow	pulse	0 mm	3
17	Flow	pulse	2 mm	3
18	Flow	pulse	4 mm	3
19	Evetric	standard	0 mm	3
20	Evetric	standard	2 mm	3
21	Evetric	standard	4 mm	3
22	Evetric	soft start	0 mm	3
23	Evetric	soft start	2 mm	3
24	Evetric	soft start	4 mm	3
25	Evetric	pulse	0 mm	3
26	Evetric	pulse	2 mm	3
27	Evetric	pulse	4 mm	3

**Table 3:** Results of comparisons of the degree of conversion at the top and bottom of samples according to the evaluated parameters and their interactions.

	Degree of Conversion—Top of the Sample		Degree of Conversion—Bottom of the Sample	
	F	*p*-Value	F	*p*-Value
Material	183.59	<0.001 *	68.56	<0.001 *
Exposure mode	0.96	0.391	3.66	0.032 *
Distance	3.20	0.049 *	2.83	0.068
Material × Exposure mode	0.08	0.988	1.30	0.280
Material × Distance	0.60	0.665	0.61	0.656
Exposure mode × Distance	1.38	0.252	1.71	0.161
Material × Exposure mode × Distance	1.10	0.380	1.23	0.298

* significant differences.

## Data Availability

The original contributions presented in this study are included in the article/Supplementary Material. Further inquiries can be directed to the corresponding author.

## Supplementary Materials

The following supporting information can be downloaded at https://www.mdpi.com/article/10.3390/ma19071390/s1. Table S1: Results of Tukey post hoc test comparisons. Table S2: The parameters of Lorentzian peaks obtained in spectra deconvolution shown in Figure 2. FWHM is Full Width at Half Maximum.

## References

[1] Lehmann A., Nijakowski K., Jankowski J., Donnermeyer D., Palma P.J., Drobac M., Martins J.F.B., Pertek Hatipoğlu F., Tulegenova I., Javed M.Q. (2024). Awareness of Possible Complications Associated with Direct Composite Restorations: A Multinational Survey among Dentists from 13 Countries with Meta-Analysis. J. Dent..

[2] Barreto Girão L., Ohana de Lima Martins J., Lemos J.V.M., Pinto M.R., Rolim J.P.M.L., Alves e Silva F.C.F., Saboia V.d.P.A., Bitu Sousa F., de Barros Silva P.G. (2020). Influence of the Degree of Conversion and Bis-GMA Residues of Bulk Fill Resins on Tissue Toxicity in an Subcutaneous Model in Rats. J. Appl. Biomater. Funct. Mater..

[3] Beriat N.C., Ertan A.A., Canay S., Gurpinar A., Onur M.A. (2010). Effect of Different Polymerization Methods on the Cytotoxicity of Dental Composites. Eur. J. Dent..

[4] Lehmann A., Nijakowski K., Jankowski J., Donnermeyer D., Ramos J.C., Drobac M., Martins J.F.B., Hatipoğlu Ö., Omarova B., Javed M.Q. (2025). Clinical Difficulties Related to Direct Composite Restorations: A Multinational Survey. Int. Dent. J..

[5] Najjar Y.M., Burhan A.S., Hajeer M.Y., Nawaya F.R. (2023). Effects of the Conventional, Soft Start, and Pulse Delay Modes Produced by Light-Emitting Diode Device on Microleakage beneath Metal Brackets: An in Vitro Comparative Study. Int. Orthod..

[6] Hatipoğlu Ö., Martins J.F.B., Karobari M.I., Taha N., Aldhelai T.A., Ayyad D.M., Madfa A.A., Martin-Biedma B., Fernandez R., Omarova B.A. (2024). Repair versus Replacement of Defective Direct Dental Restorations: A Multinational Cross-Sectional Study with Meta-Analysis. J. Dent..

[7] Aqili T.M., Hakeem M.M., Almahmadi D.S., Alahmadi R.B., Alsani A.A., Binmahmoud S.M., Alotaibi W.K., Mirah M. (2024). Pulpitis Tendency in Teeth with Vital Pulp Restored with Composite Resins: A Cross-Sectional Retrospective Study. Int. J. Community Med. Public Health.

[8] Lim H.-K., Keerthana S., Song S.-Y., Li C., Shim J.S., Ryu J.J. (2024). Effect of Light Irradiance and Curing Duration on Degree of Conversion of Dual-Cure Resin Core in Various Cavities with Different Depths and Diameters. Materials.

[9] Paolone G., Pavan F., Guglielmi P.C., Scotti N., Cantatore G., Vichi A. (2022). In Vitro Procedures for Color Stability Evaluation of Dental Resin-Based Composites Exposed to Smoke: A Scoping Review. Dent. Mater. J..

[10] Roque-Trujillo J.F., Murillo-Gómez F., Roque-Trujillo J.F., Murillo-Gómez F. (2025). Influence of the Cavity-Depth/Light Tip-Material Distance on the Degree of Conversion and Physical Properties of a Nanohybrid Resin Composite Employing the Incremental Technique. Odovtos Int. J. Dent. Sci..

[11] Al Nahedh H., Al-Senan D.F., Alayad A.S. (2022). The Effect of Different Light-Curing Units and Tip Distances on the Polymerization Efficiency of Bulk-Fill Materials. Oper. Dent..

[12] Mahn E. (2013). Clinical Criteria for the Successful Curing of Composite Materials. Rev. Clínica Periodoncia Implantol. Rehabil. Oral.

[13] Al-Senan D., Al-Nahedh H. (2022). The Effect of Different Light Curing Units and Tip Distances on Translucency Parameters of Bulk Fill Materials. Saudi Dent. J..

[14] Yilmaz F. (2025). Predictors and Development Patterns of Approximal Caries: A Retrospective Cross-Sectional Radiographic Assessment. BMC Oral Health.

[15] Deger C., Senol A.A., Yılmaz Atalı P., Oglakci Ozkoc B. (2025). Interfacial Gap Formation of Class II Composite Restorations with Proximal Box Elevation Using Bulk-Fill Materials: A Micro-CT Study. Sci. Rep..

[16] Kopperud S.E., Espelid I., Tveit A.B., Skudutyte-Rysstad R. (2015). Risk Factors for Caries Development on Tooth Surfaces Adjacent to Newly Placed Class II Composites—A Pragmatic, Practice Based Study. J. Dent..

[17] Nedeljkovic I., De Munck J., Vanloy A., Declerck D., Lambrechts P., Peumans M., Teughels W., Van Meerbeek B., Van Landuyt K.L. (2020). Secondary Caries: Prevalence, Characteristics, and Approach. Clin. Oral Investig..

[18] Albelasy E.H., Hamama H.H., Chew H.P., Montaser M., Mahmoud S.H. (2022). Secondary Caries and Marginal Adaptation of Ion-Releasing versus Resin Composite Restorations: A Systematic Review and Meta-Analysis of Randomized Clinical Trials. Sci. Rep..

[19] dos Santos Sousa G., Guimarães G.F., Marcelino E., Rodokas J.E.P., de Oliveira Júnior A.J., Cesarino I., Leão A.L., dos Santos Riccardi C., Arjmand M., Simões R.P. (2021). Shrinkage Stress and Temperature Variation in Resin Composites Cured via Different Photoactivation Methods: Insights for Standardisation of the Photopolymerisation. Polymers.

[20] Poggio C., Lombardini M., Gaviati S., Chiesa M. (2012). Evaluation of Vickers Hardness and Depth of Cure of Six Composite Resins Photo-Activated with Different Polymerization Modes. J. Conserv. Dent. JCD.

[21] Lehmann A., Nijakowski K., Mroczyk M., Podgórski F., Czarnecka B., Surdacka A. (2024). Influence of Exposure Distance on Light Irradiance of Dental Curing Lamps in Various Operating Modes. Appl. Sci..

[22] Ozciftci G., Boyacioglu H., Turkun L.S. (2025). Degree of Conversion and Microhardness of Different Composite Resins Polymerized with an Advanced LED-Curing Unit. BMC Oral Health.

[23] Abed Y.A., Sabry H.A., Alrobeigy N.A. (2015). Degree of Conversion and Surface Hardness of Bulk-Fill Composite versus Incremental-Fill Composite. Tanta Dent. J..

[24] Lempel E., Őri Z., Szalma J., Lovász B.V., Kiss A., Tóth Á., Kunsági-Máté S. (2019). Effect of Exposure Time and Pre-Heating on the Conversion Degree of Conventional, Bulk-Fill, Fiber Reinforced and Polyacid-Modified Resin Composites. Dent. Mater. Off. Publ. Acad. Dent. Mater..

[25] Wojdyr M. (2010). Fityk: A General-Purpose Peak Fitting Program. J. Appl. Crystallogr..

[26] Babaei B., Cella S., Farrar P., Prentice L., Prusty B.G. (2022). The Influence of Dental Restoration Depth, Internal Cavity Angle, and Material Properties on Biomechanical Resistance of a Treated Molar Tooth. J. Mech. Behav. Biomed. Mater..

[27] AlSheikh R., Almulhim K.S., Abdulkader M., Haridy R., Bugshan A.S., Aldamanhouri R., Elgezawi M. (2022). Toward a Clinically Reliable Class II Resin Composite Restoration: A Cross-Sectional Study into the Current Clinical Practice among Dentists in Saudi Arabia. Int. J. Dent..

[28] Gatin E., Iordache S.-M., Matei E., Luculescu C.-R., Iordache A.-M., Grigorescu C.E.A., Ilici R.R. (2022). Raman Spectroscopy as Spectral Tool for Assessing the Degree of Conversion after Curing of Two Resin-Based Materials Used in Restorative Dentistry. Diagnostics.

[29] Chrószcz M.W., Barszczewska-Rybarek I.M., Wori P. (2021). The Relationship between the Degree of Conversion in Dental Dimethacrylate Polymers Determined by Infrared Spectroscopy and Polymerization Shrinkage. Eng. Proc..

[30] Santini A., Miletic V., Koutsaki D. (2012). Degree of Conversion of Three Fissure Sealants Cured by Different Light Curing Units Using Micro-Raman Spectroscopy. J. Dent. Sci..

[31] Acquaviva P.A., Cerutti F., Adami G., Gagliani M., Ferrari M., Gherlone E., Cerutti A. (2009). Degree of Conversion of Three Composite Materials Employed in the Adhesive Cementation of Indirect Restorations: A Micro-Raman Analysis. J. Dent..

[32] Conti C., Giorgini E., Landi L., Putignano A., Tosi G. (2005). Spectroscopic and Mechanical Properties of Dental Resin Composites Cured with Different Light Sources. J. Mol. Struct..

[33] Néma V., Kunsági-Máté S., Őri Z., Kiss T., Szabó P., Szalma J., Fráter M., Lempel E. (2024). Relation between Internal Adaptation and Degree of Conversion of Short-Fiber Reinforced Resin Composites Applied in Bulk or Layered Technique in Deep MOD Cavities. Dent. Mater..

[34] Ozturk B., Cobanoglu N., Cetin A.R., Gunduz B. (2013). Conversion Degrees of Resin Composites Using Different Light Sources. Eur. J. Dent..

[35] Javed F., Tewari R.K., Alam S., Husain S., Hasan F. (2025). Effect of Different Light-Curing Modes and Curing Times on Degree of Conversion and Microhardness of Three Different Bulk-Fill Composites: An in Vitro Study. Clin. Oral Investig..

[36] Oglakci B., Enginler-Özlen R.H., Demirkol M., Özduman Z.C., Kucukyildirim B.O., Eliguzeloglu-Dalkilic E., Oglakci B., Enginler-Özlen R.H., Demirkol M., Özduman Z.C. (2022). The Effect of Curing Modes and Times of Third-Generation Led LCU on the Mechanical Properties of Nanocomposites. Odovtos Int. J. Dent. Sci..

[37] Barcelos L.M., Braga S., Pereira R., Price R.B., Soares C.J. (2023). Effect of Using Manufacturer-Recommended Exposure Times to Photo-Activate Bulk-Fill and Conventional Resin-Based Composites. Oper. Dent..

[38] Giannini M., de Castro E.F., Sahadi B.O., Albuquerque R.d.C., Rueggeberg F.A. (2025). Composite Monomer Conversion and Microhardness Using Short Curing Times. Braz. Dent. J..

[39] Lempel E., Czibulya Z., Kovács B., Szalma J., Tóth Á., Kunsági-Máté S., Varga Z., Böddi K. (2016). Degree of Conversion and BisGMA, TEGDMA, UDMA Elution from Flowable Bulk Fill Composites. Int. J. Mol. Sci..

[40] Gömleksiz S., Gömleksiz O. (2025). Effect of Cavity Depth on Degree of Conversion and Microhardness of Low-Shrinkage Resin Composites. BMC Oral Health.

[41] Yokesh C.A., Hemalatha P., Muthalagu M., Justin M.R. (2017). Comparative Evaluation of the Depth of Cure and Degree of Conversion of Two Bulk Fill Flowable Composites. J. Clin. Diagn. Res. JCDR.

[42] Par M., Gamulin O., Marovic D., Klaric E., Tarle Z. (2015). Raman Spectroscopic Assessment of Degree of Conversion of Bulk-Fill Resin Composites—Changes at 24 Hours Post Cure. Oper. Dent..

[43] Fanfoni L., De Biasi M., Antollovich G., Di Lenarda R., Angerame D. (2020). Evaluation of Degree of Conversion, Rate of Cure, Microhardness, Depth of Cure, and Contraction Stress of New Nanohybrid Composites Containing Pre-Polymerized Spherical Filler. J. Mater. Sci. Mater. Med..

[44] Jain L., Mehta D., Meena N., Gupta R. (2018). Influence of Light Energy Density, Composite Type, Composite Thickness, and Postcuring Phase on Degree of Conversion of Bulk-Fill Composites. Contemp. Clin. Dent..

[45] Jakupović S., Pervan N., Duratbegović D., Jakupović V., Muratović E., Kobašlija S. (2025). Evaluation of the Effect of Different Light-Curing Protocols on the Microhardness of Contemporary Bulk-Fill Resin Composites. Polymers.

[46] Moldovan M., Balazsi R., Soanca A., Roman A., Sarosi C., Prodan D., Vlassa M., Cojocaru I., Saceleanu V., Cristescu I. (2019). Evaluation of the Degree of Conversion, Residual Monomers and Mechanical Properties of Some Light-Cured Dental Resin Composites. Materials.

[47] Thomaidis S., Kampouropoulos D., Antoniadou M., Kakaboura A. (2024). Evaluation of the Depth of Cure by Microhardness of Bulk-Fill Composites with Monowave and Polywave LED Light-Curing Units. Appl. Sci..

[48] Bociong K., Krasowski M., Domarecka M., Sokołowski J. (2016). Effect of the Method of Photopolymerization of Dental Composites Based on Dimethacrylate Resin on the Shrinkage Stresses and Selected Properties of the Cured Material. Polimery.

[49] Bardocz-Veres Z., Miklós M.L., Biró E.-K., Kántor É.A., Kántor J., Dudás C., Kerekes-Máthé B. (2024). New Perspectives in Overcoming Bulk-Fill Composite Polymerization Shrinkage: The Impact of Curing Mode and Layering. Dent. J..

[50] Halvorson R.H., Erickson R.L., Davidson C.L. (2003). The Effect of Filler and Silane Content on Conversion of Resin-Based Composite. Dent. Mater. Off. Publ. Acad. Dent. Mater..

[51] Turssi C.P., Ferracane J.L., Vogel K. (2005). Filler Features and Their Effects on Wear and Degree of Conversion of Particulate Dental Resin Composites. Biomaterials.

[52] Yu P., Yap A., Wang X.Y. (2017). Degree of Conversion and Polymerization Shrinkage of Bulk-Fill Resin-Based Composites. Oper. Dent..

[53] Szalewski L., Szalewska M., Jarosz P., Woś M., Szymańska J. (2021). Temperature Changes in Composite Materials during Photopolymerization. Appl. Sci..

[54] Janeczek M., Herman K., Fita K., Dudek K., Kowalczyk-Zając M., Czajczyńska-Waszkiewicz A., Piesiak-Pańczyszyn D., Kosior P., Dobrzyński M. (2016). Assessment of Heat Hazard during the Polymerization of Selected Light-Sensitive Dental Materials. BioMed Res. Int..

[55] Leyva del Rio D., Johnston W.M. (2023). Effect of Monomer Composition and Filler Fraction on Surface Microhardness and Depth of Cure of Experimental Resin Composites. Eur. J. Oral Sci..

