# Supplementary material for: Optimizing Dental Resin-Based Composite Polymerization with Distance and Exposure Variables: Preliminary Study
Source: Materials (Basel). 2026 Mar 31;19(7):1390. doi: 10.3390/ma19071390 (PMC13074907; doi:10.3390/ma19071390)
Supplement: Supplementary file 1 [file materials-19-01390-s001.zip › materials-4149386-supplementary.pdf]

Table S1. Results of Tukey post hoc test comparisons.

	Material	Exposure mode	Distance	1	2	3	4	5	6	7	8	9	10	11	12	13	14	15	16	17	18	19	20	21	22	23	24	25	26	27
1	Bulk fill	pulse	0 mm		1.0000	0.9919	1.0000	1.0000	0.9822	1.0000	0.9652	1.0000	0.0003	0.0002	0.0002	0.0002	0.0002	0.0002	0.0002	0.0004	0.0002	0.0005	0.0002	0.0002	0.0009	0.0002	0.0002	0.0002	0.0002	0.0002
2	Bulk fill	pulse	2 mm	0.9970		1.0000	1.0000	1.0000	0.9999	1.0000	0.9997	1.0000	0.0009	0.0003	0.0005	0.0003	0.0002	0.0002	0.0004	0.0017	0.0002	0.0024	0.0003	0.0002	0.0049	0.0002	0.0002	0.0004	0.0002	0.0003
3	Bulk fill	pulse	4 mm	0.9476	0.1036		1.0000	1.0000	1.0000	1.0000	1.0000	0.8481	0.0275	0.0034	0.0141	0.0070	0.0009	0.0009	0.0100	0.0518	0.0024	0.0700	0.0034	0.0007	0.1235	0.0003	0.0003	0.0100	0.0009	0.0049
4	Bulk fill	soft start	0 mm	0.9991	1.0000	0.1387		1.0000	0.9999	1.0000	0.9997	1.0000	0.0009	0.0003	0.0005	0.0003	0.0002	0.0002	0.0004	0.0017	0.0002	0.0024	0.0003	0.0002	0.0049	0.0002	0.0002	0.0004	0.0002	0.0003
5	Bulk fill	soft start	2 mm	1.0000	0.9991	0.9096	0.9998		1.0000	1.0000	1.0000	0.9989	0.0024	0.0004	0.0012	0.0007	0.0002	0.0002	0.0009	0.0049	0.0003	0.0070	0.0004	0.0002	0.0141	0.0002	0.0002	0.0009	0.0002	0.0005
6	Bulk fill	soft start	4 mm	0.9874	1.0000	0.0651	1.0000	0.9949		1.0000	1.0000	0.7850	0.0379	0.0049	0.0197	0.0100	0.0012	0.0012	0.0141	0.0700	0.0034	0.0935	0.0049	0.0009	0.1606	0.0004	0.0004	0.0141	0.0012	0.0070
7	Bulk fill	standard	0 mm	1.0000	1.0000	0.3730	1.0000	1.0000	1.0000		1.0000	0.9919	0.0049	0.0007	0.0024	0.0012	0.0003	0.0003	0.0017	0.0100	0.0005	0.0141	0.0007	0.0003	0.0275	0.0002	0.0002	0.0017	0.0003	0.0009
8	Bulk fill	standard	2 mm	0.8572	1.0000	0.0165	1.0000	0.9096	1.0000	0.9999		0.7123	0.0518	0.0070	0.0275	0.0141	0.0017	0.0017	0.0197	0.0935	0.0049	0.1235	0.0070	0.0012	0.2060	0.0005	0.0005	0.0197	0.0017	0.0100
9	Bulk fill	standard	4 mm	0.9949	1.0000	0.0890	1.0000	0.9983	1.0000	1.0000	1.0000		0.0002	0.0002	0.0002	0.0002	0.0002	0.0002	0.0002	0.0002	0.0002	0.0002	0.0002	0.0002	0.0003	0.0002	0.0002	0.0002	0.0002	0.0002
10	Evetric	pulse	0 mm	0.0054	0.3354	0.0002	0.2668	0.0078	0.4541	0.0890	0.7909	0.3730		1.0000	1.0000	1.0000	1.0000	1.0000	1.0000	1.0000	1.0000	1.0000	1.0000	0.9999	1.0000	0.9968	0.9968	1.0000	1.0000	1.0000
11	Evetric	pulse	2 mm	0.0095	0.4541	0.0002	0.3730	0.0137	0.5845	0.1387	0.8852	0.4969	1.0000		1.0000	1.0000	1.0000	1.0000	1.0000	1.0000	1.0000	1.0000	1.0000	1.0000	0.9999	1.0000	1.0000	1.0000	1.0000	1.0000
12	Evetric	pulse	4 mm	0.0554	0.8572	0.0003	0.7909	0.0763	0.9303	0.4541	0.9970	0.8852	1.0000	1.0000		1.0000	1.0000	1.0000	1.0000	1.0000	1.0000	1.0000	1.0000	1.0000	1.0000	0.9997	0.9997	1.0000	1.0000	1.0000
13	Evetric	soft start	0 mm	0.0010	0.1036	0.0002	0.0763	0.0014	0.1594	0.0197	0.4127	0.1201	1.0000	1.0000	0.9998		1.0000	1.0000	1.0000	1.0000	1.0000	1.0000	1.0000	1.0000	1.0000	1.0000	1.0000	1.0000	1.0000	1.0000
14	Evetric	soft start	2 mm	0.0078	0.4127	0.0002	0.3354	0.0114	0.5405	0.1201	0.8572	0.4541	1.0000	1.0000	1.0000	1.0000		1.0000	1.0000	0.9997	1.0000	0.9989	1.0000	1.0000	0.9919	1.0000	1.0000	1.0000	1.0000	1.0000
15	Evetric	soft start	4 mm	0.0651	0.8852	0.0003	0.8257	0.0890	0.9476	0.4969	0.9983	0.9096	1.0000	1.0000	1.0000	0.9995	1.0000		1.0000	0.9997	1.0000	0.9989	1.0000	1.0000	0.9919	1.0000	1.0000	1.0000	1.0000	1.0000
16	Evetric	standard	0 mm	0.0021	0.1825	0.0002	0.1387	0.0030	0.2668	0.0397	0.5845	0.2082	1.0000	1.0000	1.0000	1.0000	1.0000	1.0000		1.0000	1.0000	1.0000	1.0000	1.0000	1.0000	0.9999	0.9999	1.0000	1.0000	1.0000
17	Evetric	standard	2 mm	0.0095	0.4541	0.0002	0.3730	0.0137	0.5845	0.1387	0.8852	0.4969	1.0000	1.0000	1.0000	1.0000	1.0000	1.0000	1.0000		1.0000	1.0000	1.0000	0.9989	1.0000	0.9822	0.9822	1.0000	0.9997	1.0000
18	Evetric	standard	4 mm	0.1825	0.9874	0.0006	0.9727	0.2362	0.9970	0.7909	1.0000	0.9918	0.9999	1.0000	1.0000	0.9811	1.0000	1.0000	0.9970	1.0000		1.0000	1.0000	1.0000	0.9997	1.0000	1.0000	1.0000	1.0000	1.0000
19	Flow	pulse	0 mm	0.0763	0.9096	0.0003	0.8572	0.1036	0.9616	0.5405	0.9991	0.9303	1.0000	1.0000	1.0000	0.9991	1.0000	1.0000	1.0000	1.0000	1.0000		1.0000	0.9968	1.0000	0.9652	0.9652	1.0000	0.9989	1.0000
20	Flow	pulse	2 mm	0.0397	0.7909	0.0002	0.7134	0.0554	0.8852	0.3730	0.9918	0.8257	1.0000	1.0000	1.0000	1.0000	1.0000	1.0000	1.0000	1.0000	1.0000	1.0000		1.0000	0.9999	1.0000	1.0000	1.0000	1.0000	1.0000
21	Flow	pulse	4 mm	0.2998	0.9983	0.0012	0.9949	0.3730	0.9998	0.9096	1.0000	0.9991	0.9983	0.9998	1.0000	0.9303	0.9995	1.0000	0.9811	0.9998	1.0000	1.0000	1.0000		0.9822	1.0000	1.0000	1.0000	1.0000	1.0000
22	Flow	soft start	0 mm	0.0095	0.4541	0.0002	0.3730	0.0137	0.5845	0.1387	0.8852	0.4969	1.0000	1.0000	1.0000	1.0000	1.0000	1.0000	1.0000	1.0000	1.0000	1.0000	1.0000	0.9998		0.8994	0.8994	1.0000	0.9919	1.0000
23	Flow	soft start	2 mm	0.0114	0.4969	0.0002	0.4127	0.0165	0.6284	0.1594	0.9096	0.5405	1.0000	1.0000	1.0000	1.0000	1.0000	1.0000	1.0000	1.0000	1.0000	1.0000	1.0000	0.9999	1.0000		1.0000	0.9999	1.0000	1.0000
24	Flow	soft start	4 mm	0.0137	0.5405	0.0002	0.4541	0.0197	0.6716	0.1825	0.9303	0.5845	1.0000	1.0000	1.0000	1.0000	1.0000	1.0000	1.0000	1.0000	1.0000	1.0000	1.0000	1.0000	1.0000	1.0000		0.9999	1.0000	1.0000
25	Flow	standard	0 mm	0.0335	0.7534	0.0002	0.6716	0.0470	0.8572	0.3354	0.9874	0.7909	1.0000	1.0000	1.0000	1.0000	1.0000	1.0000	1.0000	1.0000	1.0000	1.0000	1.0000	1.0000	1.0000	1.0000	1.0000		1.0000	1.0000
26	Flow	standard	2 mm	0.0030	0.2362	0.0002	0.1825	0.0044	0.3354	0.0554	0.6716	0.2668	1.0000	1.0000	1.0000	1.0000	1.0000	1.0000	1.0000	1.0000	0.9991	1.0000	1.0000	0.9918	1.0000	1.0000	1.0000	1.0000		1.0000
27	Flow	standard	4 mm	0.0281	0.7134	0.0002	0.6284	0.0397	0.8257	0.2998	0.9811	0.7534	1.0000	1.0000	1.0000	1.0000	1.0000	1.0000	1.0000	1.0000	1.0000	1.0000	1.0000	1.0000	1.0000	1.0000	1.0000	1.0000	1.0000	

Top

Bottom

Table S2. The parameters of Lorentzian peaks obtained in spectra deconvolution shown in Figure 2. FWHM is Full Width at Half Maximum.

Top. R-squared: 0.969477

Center	Height	Area	FWHM
1638.57	34.881	542.463	9.90061
1608.05	13.7338	173.895	8.06077
1600.34	33.8171	546.75	10.2928
1588.16	17.5445	179.392	6.50941

Bottom. R-squared: 0.974491

Center	Height	Area	FWHM
1638.78	47.1931	729.655	9.84281
1608.17	14.1948	204.556	9.17411
1600.15	33.5552	556.172	10.5519
1588.13	18.7728	168.367	5.70962

Reference. R-squared: 0.981851

Center	Height	Area	FWHM
1638.74	88.6792	1277.74	9.17277
1608.74	14.3116	187.219	8.328
1600.24	29.7364	482.027	10.3196
1588.08	17.7685	156.812	5.61834
